# Peripheral Dopamine in Restless Legs Syndrome

**DOI:** 10.3389/fneur.2018.00155

**Published:** 2018-03-15

**Authors:** Ulrike H. Mitchell, J. Daniel Obray, Erik Hunsaker, Brandon T. Garcia, Travis J. Clarke, Sandra Hope, Scott C. Steffensen

**Affiliations:** ^1^Department of Exercise Sciences, Brigham Young University, Provo, UT, United States; ^2^Department of Psychology, Brigham Young University, Provo, UT, United States; ^3^Department of Microbiology and Molecular Biology, Brigham Young University, Provo, UT, United States

**Keywords:** Willis–Ekbom disease, DA, D2 receptors, white blood cells, lymphocytes, monocytes

## Abstract

**Objective/Background:**

Restless Legs Syndrome (RLS) is a dopamine-dependent disorder characterized by a strong urge to move. The objective of this study was to evalulate blood levels of dopamine and other catecholamines and blood D2-subtype dopamine receptors (D2Rs) in RLS.

**Patients/Methods:**

Dopamine levels in blood samples from age-matched unmedicated RLS subjects, medicated RLS subjects and Controls were evaluated with high performance liquid chromatography and dopamine D2R white blood cell (WBC) expression levels were determined with fluorescence-activated cell sorting and immunocytochemistry.

**Results:**

Blood plasma dopamine levels, but not norepinepherine or epinephrine levels, were significantly increased in medicated RLS subjects vs unmedicated RLS subjects and Controls. The percentage of lymphocytes and monocytes expressing D2Rs differed between Control, RLS medicated and RLS unmedicated subjects. Total D2R expression in lymphocytes, but not monocytes, differed between Control, RLS medicated and RLS unmedicated subjects. D2Rs in lymphocytes, but not monocytes, were sensitive to dopamine in Controls only.

**Conclusion:**

Downregulation of WBCs D2Rs occurs in RLS. This downregulation is not reversed by medication, although commonly used RLS medications increase plasma dopamine levels. The insensitivity of monocytes to dopamine levels, but their downregulation in RLS, may reflect their utility as a biomarker for RLS and perhaps brain dopamine homeostasis.

## Introduction

The strong urge to move is the most characteristic feature of Restless Legs Syndrome (RLS)/Willis–Ekbom Disease (WED) (1). The compulsion to move associated with this sensorimotor disorder usually follows a circadian rhythm, worsening as the day progresses and subsiding with movement (2). The pathogenesis of RLS is not clear, and several theories exist. The most compelling theory involves the neurotransmitter dopamine (DA) (3), more specifically, the hypofunctioning in brain DA signaling (4). Supporting evidence for this notion is given by the fact that RLS symptoms decrease with drugs that stimulate the DA system and are produced with drugs that block DA neurotransmission in the brain (5). The levels of DA in the brain are directly proportional to the number of brain DA D2 receptors (D2Rs) (6, 7), which are autoreceptors on DA neurons in the midbrain nigrostriatal and mesolimbic DA systems involved in reward and movement, respectively.

Dopamine is normally present at low levels (15–30 pg/ml) in human blood plasma (8). Studies have shown that in addition to spilling over from noradrenergic nerves (8), DA is also synthesized and present internally in some immune cells (9, 10). Additionally, monocytes and lymphocytes express both D1-like (Gα_s/olf_ coupled) and D2-like (Gα_i/o_ coupled) receptors (11). As such, it has been proposed that DA plays a key role in mediating a neuroimmune link (12, 13), and that peripheral DA receptor expression may be a biomarker for CNS DA function (14, 15). Consistent with the hypothesis that changes in CNS DA function affect peripheral DA receptor expression, changes in D2-like receptor expression on lymphocytes have been reported in opioid addicts (16) and in alcohol dependence (17). Further, increased D2R expression on lymphocytes has been reported in Parkinson’s disease (18). Recently D2R expression in T cells has been reported to be positively correlated with motor deficits in Parkinson’s disease (19).

An alternative theory concerning the pathogenesis of RLS involves diminished peripheral blood flow, which could also be owed to altered DA availability, albeit in the periphery. It is hypothesized that peripheral hypoxia induces the urge to move in order to improve tissue oxygenation (20–22). Blood flow is controlled by extrinsic and intrinsic factors. One main extrinsic factor is the sympathetic nervous system, which induces vasoconstriction by releasing catecholamines [i.e., norepinephrine (NE), epinephrine, and DA] (23) in the peripheral blood. It is conceivable that subjects with RLS experience augmented activity of the sympathetic nervous system, following a circadian rhythm, leading to vasoconstriction, which leaves them with tissue hypoxia at night. Other studies (24, 25) have implicated sympathetic nervous system hyperactivity in the involvement of symptoms associated with RLS.

This study was conducted to determine if there is a link between these two theories by correlating blood levels of the catecholamines NE, epinephrine (EPI), and DA to D2R expression levels on white blood cells (WBCs) in medicated and unmedicated subjects with RLS vs Controls. Knowing that these two theories are not competing, but possibly complementing each other, may help in understanding the pathogenesis of RLS.

## Materials and Methods

### Subjects

This study was carried out in accordance with the recommendations of federal ethics guidelines, Code of Federal Regulations, TITLE 45, PUBLIC WELFARE, DEPARTMENT OF HEALTH AND HUMAN SERVICES, PART 46, PROTECTION OF HUMAN SUBJECTS with written informed consent from all subjects. All subjects gave written informed consent in accordance with the Declaration of Helsinki. The protocol was approved by Institutional Review Board at the authors’ university, and each subject gave written informed consent before participation. Thirteen healthy subjects with RLS (*n* = 7 medicated; *n* = 6 unmedicated) and 12 age-matched subjects without RLS (Table 1) were recruited for this study *via* newspaper advertisement, flyer, and word of mouth. Medicated subjects were taking dopaminergic medications (*n* = 4 pramipexole; *n* = 2 ropinirole) and benzodiazepines (*n* = 1 lorazepam). The sample size was determined based on other research with a similar set up which has used the same number of subjects (26). However, no *a priori* power analysis was performed. Inclusion criteria consisted of a confirmed diagnosis of RLS using the updated International Restless Legs Syndrome Study Group consensus criteria (27) and a severity score of 11 or higher on the International RLS Study Group Rating Scale (IRLS) (28). All subjects were screened for exclusion criteria, which consisted of pregnancy and a diagnosis of iron deficiency anemia and diabetes. Candidates were also excluded if they had any known cardiovascular disease.

**Table 1:** Demographics and questionnaires.

Group	Age (years)	Women:men	Weight (kg)	Height (cm)	Years since Restless Legs Syndrome (RLS) diagnosis	IRLS score
RLS unmedicated (*n* = 6)	32.8 ± 12.1	2:1	64.8 ±14.2	172.7 ± 10.4	12.5 ± 3.5	17.3 ± 9.9
RLS medicated (*n* = 7)	57.1 ± 12.5	1:6	82.1 ± 22.3	173.4 ± 5.4	11.0 ± 2.1	22.1 ± 3.7
Control (*n* = 12)	44.5 ± 16.4	7:5	75.6 ± 14.9	172.9 ± 10.1	n/a	n/a

### Sample Collection and Extraction of Blood

Blood samples for the determination of catecholamine and for D2R flow were collected between 1800–2100 hours in all subjects to control for diurnal variations. For blood collection, an antecubital catheter was implanted in a median cubital vein and remained *in situ* for 20 min to control for the stress of the injection. On average 6 mL of blood was drawn in heparinized tubes with a 100-µL aliquot of blood being set aside for cytometry (see D2R Flow Cytometry).

#### Measurement of Catecholamines

The whole blood sample was immediately centrifuged (2,000 rpm) at 4°C for 10 min and the plasma collected for catecholamine analysis. The plasma samples were snap frozen in liquid nitrogen and then sent frozen to Labcorp. Labcorp used High-Pressure Liquid Chromatography with electrochemical detection for determination of catecholamine levels.

#### D2R Flow Cytometry

A 100 µL aliquot of whole blood was placed in red blood cell lysis solution (BioLegend, 2.0 mL) for 10 min. The reaction was stopped with the addition of 2.0 mL PBS and centrifuged (2,000 rpm) for 5 min. Afterward, the supernatant was decanted and the leftover pellet was suspended in Cell Staining Buffer (Biolegend) for 15 min. Following an additional centrifugation step, the cell samples were incubated for 15 min on ice with anti-D2R (Millipore) antibodies. The samples were then centrifuged again and incubated for 15 min with anti-CD45 (Invitrogen APC Conjugated, 1:150) and FITC secondary (BD biosciences, 1:150) in the dark and on ice. Sample preparation was finished with an additional centrifugation step and the cells were suspended in 300 µL of cell staining buffer prior to fluorescence-activated cell sorting (FACS) on an Attune Acoustic Focusing Flow Cytometer (Applied Biosystems). Residual samples were plated onto microscope slides and treated with DAPI nuclear stain for visual confirmation of D2R expression on WBCs.

#### Incubation of WBCs With Dopamine

Blood was drawn from four separate volunteers (male, college aged with no association with RLS) and cultured to determine the effects of DA on D2R expression. An average of 10 mL of whole blood were drawn from the subjects at midday without use of the antecubital catheters. The whole blood was then mixed with equal amounts of Hank’s solution and layered on top of lymphocyte separation media (Cellgro) for separation of lymphocytes and monocytes from other whole blood constituents. The samples were then centrifuged (1,200 rpm, 4 C) for 15 min with no brake. Following centrifugation, the lymphocyte/monocyte layer of cells were extracted from the fractionated samples and then added to 10 mL of 0.1 M PBS and centrifuged (2,000 rpm, 4 C) again for 5 min. The resulting pellet was suspended in 24 mL cell culture media (DMEM).

The samples were dispersed onto 12-well plates and additional media was added which contained DA. The final volume of each well was 2.0 mL and the concentrations of DA were 0, 0.001, 0.1, 1, and 10 µM (each set was completed in triplicate). One plate was immediately harvested for flow cytometric analysis, one plate was allowed to incubate (37°C, 5% CO_2_) for an hour, and a final plate was allowed to incubate for 24 h. Following the incubation period, the cells were mechanically harvested for FACS (see above for description of staining procedure).

#### Data Analysis: Catecholamines and D2R Expression

Blood plasma catecholamine levels and D2R expression in WBCs are presented as mean ± SEM and analyzed by ANOVA. The percentage of cells expressing D2Rs and total D2R expression (as measured by mean fluorescence intensity) were compared in monocytes and lymphocytes between subjects with RLS and medicated, with RLS and unmedicated and without RLS. Statistical significance was considered at *p* < 0.05. Prior to analysis by ANOVA, data were checked for outliers by calculating the median and interquartile range (IQR). A datum was considered to be an outlier if it was larger or smaller than the median plus or minus three IQR. Using this method, two outlying datapoints were identified. After determining that the outliers were not the result of data entry error the values were bounded to the predetermined limit for classifying outliers (median ± 3 IQR). Where the ANOVA detected a significant effect between subjects a *post hoc* test with a Bonferroni correction was performed to further elucidate the nature of the effect. Statistical analysis was carried out using Stata 14.0 (StataCorp LLC, College Station, TX, USA). At the request of a reviewer a Kruskal–Wallis test was also performed at a later date. Where significance was indicated by the Kruskal–Wallis test a Conover-Iman *post hoc* test with a Bonferroni correction was performed to allow for a more complete explanation of the effect. The results for the nonparametric test are reported immediately after the results for the parametric test where appropriate.

#### Data Analysis: Effect of DA on D2R Expression

Data are presented as mean ± SEM and analyzed by ANOVA with the concentration of DA during the incubation phase as the between groups factor. Following analysis by ANOVA, a *post hoc* analysis with Bonferroni correction was performed comparing lymphocyte expression of D2Rs at each DA concentration to D2R expression in lymphocytes not incubated in DA. Repeated measures ANOVAs had revealed no effect of time on the measures of D2R expression in monocytes and lymphocytes. As such, data for D2R expression in monocytes and lymphocytes not incubated in DA were collapsed across the 0 and 1 h timepoints for this analysis. Statistical significance was considered at *p* < 0.05. Data were screened prior to analysis as described above. Statistical analysis of data was carried out using SPSS 11.0 (Armonk, NY, USA) and Stata 14.0 (StataCorp LLC, College Station, TX, USA). At the request of a reviewer a Kruskal–Wallis test was also performed at a later date. Where significance was indicated by the Kruskal–Wallis test a Conover-Iman *post hoc* test with a Bonferroni correction was performed to allow for a more complete explanation of the effect. The results for the nonparametric test are reported immediately after the results for the parametric test where appropriate.

## Results

### Blood Catecholamine Levels in RLS

**Figure 1:**
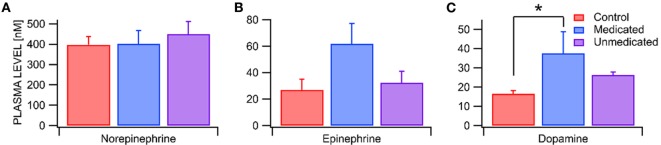
Enhanced dopamine levels in restless legs syndrome (RLS) medicated subjects. **(A)** Blood plasma norepinepherine (NE) levels were the highest of all the catecholamines, but did not differ between Control and RLS medicated and unmedicated subjects. **(B)** Epinepherine (EPI) levels likewise did not differ between Control and RLS medicated and unmedicated subjects. **(C)** Dopamine (DA) levels were significantly greater in RLS medicated subjects than in Controls. Asterisk * indicates significance level *p* < 0.05.

We compared the plasma levels of the catecholamines NE, EPI, and DA in RLS unmedicated, RLS medicated and control subjects. While plasma NE and EPI levels did not differ between RLS unmedicated, RLS medicated, and control subjects [RLS unmedicated NE = 450 ± 62 pg/mL vs RLS medicated NE = 402 ± 66 pg/mL vs Control NE = 398 ± 39 pg/mL, *F*_(2,15)_ = 0.24, *p* = 0.79, *n* = 4, 5, 9, χ^2^_(2,_ *_n_*_=18)_ = 1.14, *p* = 0.57; RLS unmedicated EPI = 32 ± 9 pg/mL vs RLS medicated EPI = 62 ± 15 pg/mL vs Control NE = 27 ± 8 pg/mL, *F*_(2,15)_ = 2.94, *p* = 0.08, *n* = 4, 5, 9 each, $\chi_{\left(2,n=18\right)}^{2}=4.32$, *p* = 0.12], there was a significant effect of condition on plasma DA levels (RLS unmedicated DA = 26 ± 2 pg/mL vs RLS medicated DA = 38 ± 11 pg/mL vs Control DA = 17 ± 2 pg/mL, *F*_(2,15)_ = 3.84, *p* = 0.05, *n* = 4, 5, 9, $\chi_{\left(2,n=18\right)}^{2}=3.56$, *p* = 0.17 each; Figures 1A–C). *Post hoc* analysis with a Bonferroni correction revealed that plasma DA levels were significantly elevated in RLS medicated but not RLS unmedicated subjects as compared with control subjects [RLS medicated vs Control *t*_(15)_ = 2.76, *p* = 0.015; RLS unmedicated vs Control *t*_(15)_ = 1.18, *p* = 0.257].

### Blood Dopamine D2R Expression in RLS

**Figure 2:**
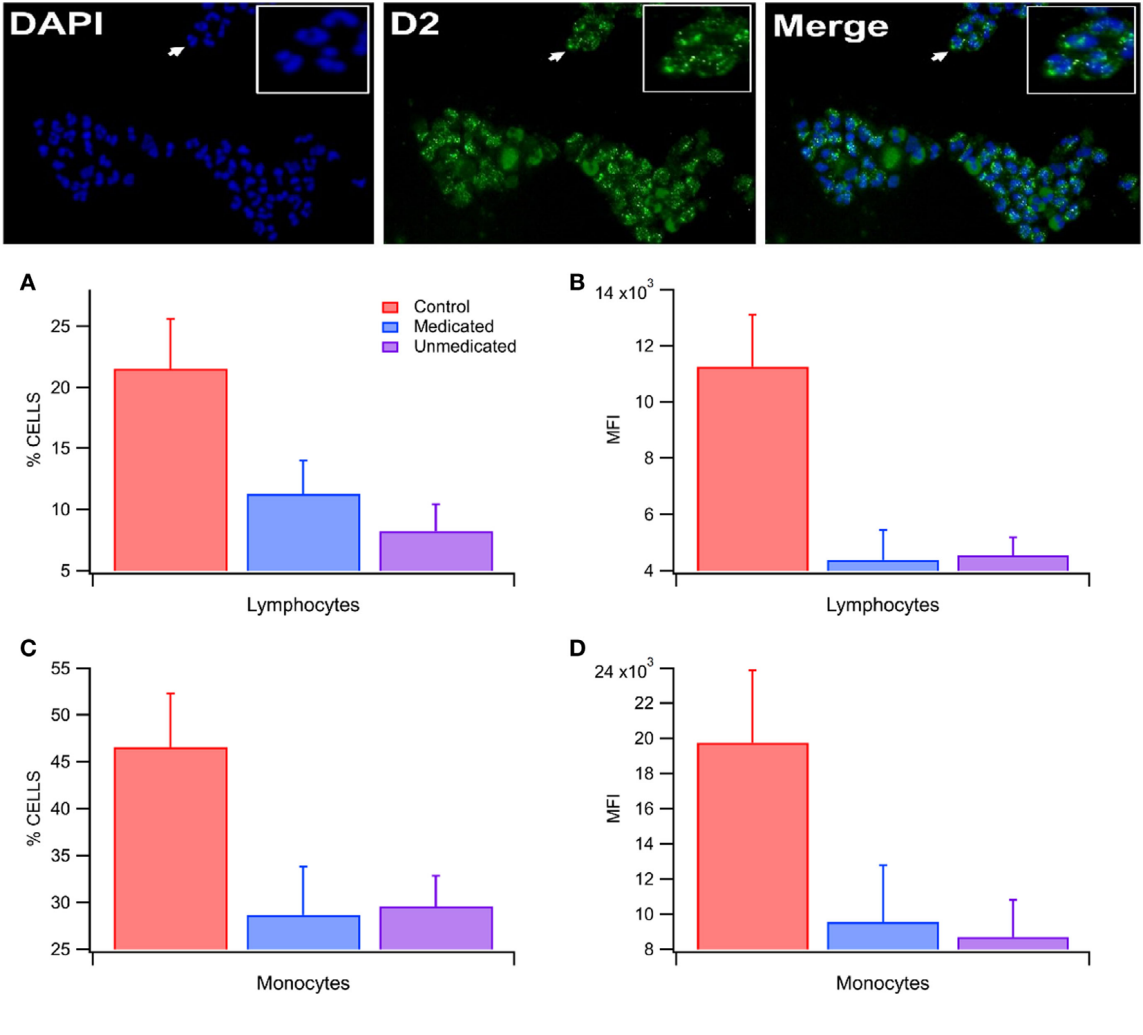
Lowered blood D2-subtype dopamine receptor (D2R) expression in restless legs syndrome (RLS). (Top) This immunocytochemistry panel of isolated white blood cells (WBCs) shows DAPI nuclear stain (blue), D2Rs (green) and merge from a control subject. Note the punctate expression of D2Rs in many WBCs. Inset is magnified view of area denoted by arrows. **(A)** Percent expression of D2Rs in lymphocytes in Control, RLS medicated, and RLS unmedicated subjects. The percent of lymphocytes expressing D2Rs differs between subjects in the different populations with expression that is lower in RLS medicated and unmedicated subjects. **(B)** The total expression of D2Rs in lymphocytes differs between the different subject populations. RLS medicated and unmedicated subjects have lower total expression of D2Rs in lymphocytes. **(C)** Likewise, the percent of monocytes expressing D2Rs differs among subject populations with expression that is lower in RLS medicated and unmedicated subjects. **(D)** The total expression of D2Rs in monocytes was not detectably different between the different subject populations. Asterisks *, ** indicate significance levels *p* < 0.05 and *p* < 0.01, respectively.

D2-subtype dopamine receptors were expressed in both lymphocytes and monocytes in both RLS and control subjects (Figure 2). In control subjects, the % cells expressing D2Rs and the total expression of D2Rs/cell was significantly greater in monocytes than lymphocytes [% cells: 49.6 ± 5.0 vs 13.3 ± 1.5; *F*_(1,43)_ = 48.9, *p* = 1.49E−08; total expression: 16,192.1 ± 2,020.3 vs 4,078.8 ± 508.5; *F*_(1,43)_ = 33.8, *p* = 7.34E−07; *n* = 22 each; Figure 2D and 2B]. The % cells expressing D2Rs in lymphocytes was significantly different among RLS unmedicated, RLS medicated and control subjects [*F*_(2,22)_ = 3.69, *p* = 0.04; *n* = 25; *n* = 6, 7, 12, $\chi_{\left(2,\;n=25\right)}^{2}=4.82$, *p* = 0.09; Figure 2A], as was the % cells expressing D2Rs in monocytes [*F*_(2,22)_ = 3.64, *p* = 0.04; *n* = 6, 7, 12, $\chi_{\left(2,\;n=25\right)}^{2}=5.01$, *p* = 0.08; Figure 2C]. A follow up analysis using Bonferroni corrected *post hoc* tests failed to detect significant differences in D2R expression between RLS unmedicated [*t*_(22)_ = −2.44, *p* = 0.07; *t*_(22)_ = −2.09, *p* = 0.14] and RLS medicated subjects [*t*_(22)_ = −1.98, *p* = 0.18; *t*_(22)_ = −2.33, *p* = 0.09] and control subjects for % cells expressing D2Rs in lymphocytes and monocytes, respectively. The total expression of D2Rs in lymphocytes was also significantly different among RLS unmedicated, RLS medicated and control subjects [*F*_(2,22)_ = 6.28, *p* = 0.01; *n* = 6, 7, 12, $\chi_{\left(2,\;n=25\right)}^{2}=9.36$, *p* < 0.01; Figure 2B], whereas the total expression of D2Rs in monocytes did not differ across groups [*F*_(2,22)_ = 2.70, *p* = 0.09; *n* = 6, 7, 12, $\chi_{\left(2,\;n=25\right)}^{2}=5.56$, *p* = 0.06; Figure 2D]. A Bonferroni corrected *post hoc* test revealed that both RLS unmedicated [*t*_(22)_ = −2.80, *p* = 0.03, *t* = 2.78, *p* = 0.02] and RLS medicated [*t*_(22)_ = −3.02, *p* = 0.02, *t* = 3.34, *p* < 0.01] subjects differed significanlty from control subjects for total expression of D2Rs in lymphocytes.

### Effects of Dopamine on Blood WBC D2R Expression

**Figure 3:**
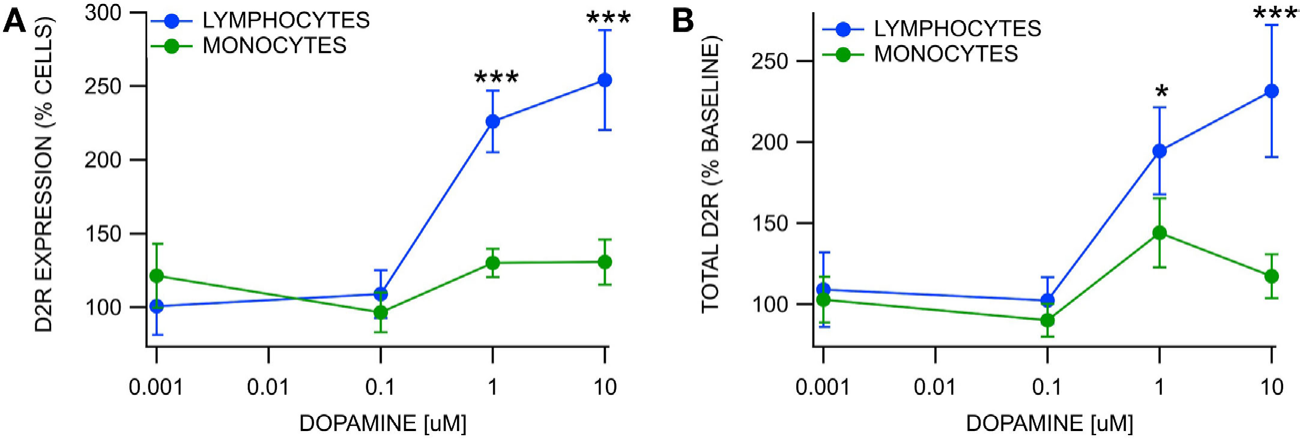
Differential upregulation of D2 receptors in white blood cells by dopamine. **(A)** Percent expression of D2-subtype dopamine receptors (D2Rs) in blood lymphocytes vs monocytes following a 1-h incubation with DA at varying concentrations. While the % of monocytes expressing D2Rs was not affected by DA, the % of lymphocytes expressing D2Rs was significantly enhanced at 1.0 and 10 µM DA. **(B)** While the total expression of D2Rs in monocytes was not affected by DA, the total expression of D2Rs in lymphocytes was significantly enhanced at 1.0 and 10 µM DA. Asterisks *, *** indicate significance levels *p* < 0.05 and *p* < 0.001, respectively.

We evaluated D2R expression in lymphocytes and monocytes at DA concentration levels 0.0, 0.001, 0.1, 1.0, and 10.0 µM following a 1-h incubation (Figure 3). The % cells expressing D2Rs in monocytes was unaffected by DA at the 0.001–10.0 µM levels compared to 0.0 µM [*F*_(4,51)_ = 1.91, *p* > 0.05; *n* = 7, 7, 10, 10, 10, $\chi_{\left(4,\;n=56\right)}^{2}=7.11$, *p* = 0.13; Figure 3A], but the % cells expressing D2Rs in lymphocytes was significantly increased at the 1.0 and 10.0 µM levels [overall: *F*_(4,51)_ = 18.69, *p* < 0.01, $\chi_{\left(4,\;n=56\right)}^{2}=31.22$, *p* < 0.01; pairwise:1.0 μM: *t*_(51)_ = 4.64, *p* < 0.01, *t* = −5.25, *p* < *0.01*; 10.0 μM: *t*_(51)_ = 7.53, *p* < 0.01; *n* = 7, 7, 10, 10, 10, *t* = −6.69, *p* < 0.01; Figure 3A]. The total expression of D2Rs in monocytes was unaffected by DA at 0.001–10.0 µM levels [*F*_(4,51)_ = 1.42, *p* > 0.05; *n* = 7, 10, 10, $\chi_{\left(4,\;n=56\right)}^{2}=6.26$, *p* = 0.18; Figure 3B], but the total expression of lymphocytes was significantly increased at the 1.0 and 10.0 µM levels [overall: *F*_(4,51)_ = 8.35, *p* < 0.01, $\chi_{\left(4,\;n=56\right)}^{2}=22.41$, *p* < 0.01; pairwise:1.0 μM: *t*_(51)_ = 2.73, *p* = 0.04, *t* = −3.72, *p* < 0.01; 10.0 μM: *t*_(51)_ = 5.18, *p* < 0.01, *t* = −5.05, *p* < 0.01; *n* = 10, 10; Figure 3B].

## Discussion

Results from both sets of analyses are included in the results section to provide readers with transperancy, although only the ANOVA results will be discussed. This is done for several reasons: (1) the data were largely normally in distributed (Shapiro–Wilk test, *p*-values > 0.05) and homoscedastic in nature (Cameron–Trivedi decomposition, *p*-values > 0.05), however, unequal in cell size. (2) A previous simulation study suggests that at a small sample size similar to that contained in our paper, with similar properties, the Kruskal–Wallis test does indeed reduce the type I error rate (approximately 2%) as compared with an ANOVA *F*-test. The Kruskal–Wallis test also increases the type II error rate (approximately 5%), thus rendering the ANOVA *F*-test more accurate (29). Further, at a slightly larger sample size similar to that contained in our DA effects on WBC D2R expression experiments, the type I and type II error rate were found to be lower for the ANOVA *F*-test than for the Kruskal–Wallis test (29). The Kruskal–Wallis test was included as a courtesy to readers at the request of a reviewer. We recognize that in three instances the results of the Kruskal–Wallis test did not reach significance where the ANOVA *F*-test did. This may be due to either Type I error on the part of the ANOVA *F*-test or type II error on the part of the Kruskal–Wallis test. Without increased sample size it is difficult to determine which is the case. We welcome future studies with improved sample size that will help to clarify this question.

### Blood Catecholamine Levels in RLS

In an attempt to consolidate the two major theories regarding the etiology of RLS, we evaluated peripheral DA levels and their target D2Rs on WBCs in RLS vs control subjects. We found that DA levels (but not NE or EPI) were significantly increased in RLS medicated subjects, but not in non-medicated subjects, vs Controls. The medications most commonly used to treat RLS symptoms are DAergic drugs, such as levodopa and DA agonists (30). Our RLS medicated subjects exhibited on average more than twice the amount of DA compared to Controls. While intake of DA or DAergic drugs might be necessary to treat a hypoDAergic state in the brain, it appears that it also leads to an increase in amounts of peripheral DA. At physiological levels DA acts as a potent vasodilator in systemic arteries (31) and enhances blood flow in peripheral skeletal muscles by inhibiting sympathetic vasoconstrictor tone (32). The administration of additional DA in the peripheral blood could be one of the ways that DAergic drugs help decrease symptoms associated with RLS. Supporting this hypothesis are recent findings, where our lab showed that by increasing blood flow in the legs through intermittent whole-body vibration (33) the symptoms of RLS are decreased. Several other blood flow augmenting modalities (21, 22, 34–37) have been linked to a decrease of symptoms associated with RLS.

### Blood Dopamine D2R Expression in RLS

Our findings indicate that medicated and unmedicated subjects with RLS exhibit a lower percentage of lymphocytes and monocytes expressing D2Rs and lower total expression of D2R in lymphocytes. This is the first study assessing D2Rs in peripheral blood in subjects with RLS, so no comparisons can be made. It is interesting to note that in postmortem tissue, decreases in D2R expression in the putamen have been found in RLS patients, with decreases in D2R expression correlating with increased severity of RLS symptoms (38). Furthermore, lower D2R binding potentials have been reported in the caudate and putamen of patients with RLS (39). Thus, if RLS is the result of a hypoDAergic state, accompanied by decreased D2R expression in the caudate, DAergic medication may help people with RLS by decreasing neuronal excitability in the nigrostriatal pathway (40), resulting in decreased RLS symptoms.

### Effects of Dopamine on Blood WBC D2R Expression

D2-subtype dopamine receptor expression in lymphocytes, but not monocytes, was sensitive to DA in control subjects. Lymphocytic D2Rs were found to adapt fairly rapidly to varying levels of circulating DA, but monocytes appeared to be insensitive to them. Specifically, lymphocytes responded to incubation in 1.0 and 10.0 µM concentrations by increasing both the total expression of D2Rs and the number of cells expressing D2Rs. Monocytes did not change expression of D2Rs following incubation in concentrations of DA ranging from 0.001 to 10.0 µM. This finding is consistent with previous findings that DA receptor activity in lymphocytes, but not monocytes modulates tyrosine hydroxylase activity (9), suggesting that DA receptors on lymphocytes and monocytes may differ in DA signal transduction. Additionally, the relative insensitivity of monocyte and lymphocyte D2R expression to DA suggests that changes in D2R expression in RLS patients are not due to changes in blood DA concentrations but may result from changes in CNS DA function, suggesting that monocyte and lymphocyte D2R expression may be a useful biomarker of CNS DA function as well as RLS. The insensitivity of monocytes to DA levels, in connection with their downregulation in RLS, may reflect their utility as a biomarker for RLS, DA-dependent disorders and perhaps brain DA homeostasis.

## Conclusion

Downregulation of WBCs D2Rs occurs in medicated and unmedicated subjects with RLS. Hence, the downregulation is not reversed by medication, although commonly used RLS medications increase plasma dopamine levels.

The insensitivity of monocytes to dopamine levels, combined with their downregulation in RLS, may reflect their utility as a biomarker for RLS and perhaps brain dopamine homeostasis.

## Ethics Statement

This study was carried out in accordance with the recommendations of federal ethics guidelines, Code of Federal Regulations, TITLE 45, PUBLIC WELFARE, DEPARTMENT OF HEALTH AND HUMAN SERVICES, PART 46, PROTECTION OF HUMAN SUBJECTS with written informed consent from all subjects. All subjects gave written informed consent in accordance with the Declaration of Helsinki. The protocol was approved by Institutional Review Board at the authors’ university, and each subject gave written informed consent before participation.

## Author Contributions

UM: study concept, data collection, data analysis, writing of manuscript, and funding. JO: data analysis and writing of manuscript. EH: data collection and writing of manuscript. BG: data collection, data analysis, and writing of manuscript. TC: data collection and data analysis. SH: data analysis and writing of manuscript. SCS: study concept, data analysis, writing of manuscript, and funding.

## Conflict of Interest Statement

All authors declare that this work was not carried out in the presence of any personal, professional, or financial relationships that could potentially be construed as a conflict of interest.
